# Determinants of breastfeeding practices in Myanmar: Results from the latest nationally representative survey

**DOI:** 10.1371/journal.pone.0239515

**Published:** 2020-09-24

**Authors:** Yadanar, Kyaw Swa Mya, Nopphol Witvorapong

**Affiliations:** 1 Department of Public Health Laboratory Science, University of Public Health, Yangon, Myanmar; 2 Department of Biostatistics and Medical Demography, University of Public Health, Yangon, Myanmar; 3 Center for Health Economics, Faculty of Economics, Chulalongkorn University, Bangkok, Thailand; Tulane University School of Public Health and Tropical Medicine, UNITED STATES

## Abstract

Optimal breastfeeding practices can ensure healthy growth and development of infants, which in the long term can impact the country's economic development. Nevertheless, Myanmar has yet to achieve the WHO’s target of 70% for early initiation of breastfeeding, and the country’s target of 90% for exclusive breastfeeding. The purpose of this study was to assess the associations between early initiation of breastfeeding and exclusive breastfeeding and bio-demographic, socio-economic and behavioral factors in Myanmar. Using the 2015–2016 Myanmar Demographic and Health Survey, the analysis of early initiation of breastfeeding was based on a sample of 1,506 under-2 children and the analysis of exclusive breastfeeding was based on a sample of 376 children aged 0–5 months. Multiple logistic modeling, with heteroskedasticity-adjusted standard errors, was used. The prevalence rates of early initiation of breastfeeding and exclusive breastfeeding in the study were 67.9% and 52.2% respectively. Having a vaginal delivery (AOR = 2.5; 95% CI = 1.7–3.7) and having frequent (≥ 4) antenatal visits (AOR = 2.4; 95% CI = 1.5–3.8) were associated with higher odds of early initiation of breastfeeding. Having a postnatal checkup (AOR = 0.5; 95% CI = 0.3–0.9) and having an infant that was perceived to be small at birth (AOR = 2.5; 95% CI = 1.1–5.7, for infants perceived to be large at birth) were significantly associated with decreased odds of exclusive breastfeeding. In order to promote optimal breastfeeding practices, this study suggested that delivery and quality of health services during pregnancy need to be strengthened in Myanmar.

## Introduction

Early initiation of breastfeeding (EIBF) is defined as breastfeeding within the first hour after giving birth. Exclusive breastfeeding (EBF) is defined as the exclusive provision of breast milk (without water, other liquids or milk substitutes, with the exception of oral rehydrated solution, drops of vitamins, minerals or medicines) to infants aged between 0–5 months. Both EIBF and EBF represent important infant and young child feeding (IYCF) indicators, according to the WHO [1–4].

Suboptimal breastfeeding practices have adverse consequences particularly in low- and middle-income countries (LMICs) [5–8]. They have been associated with increased risks of neonatal [9] and under-five mortality [6, 9], accounting for more than 0.8 million deaths among children in LMICs annually [8]. They have also been linked to lower intelligence [6] and poor health outcomes later in life [6, 8] including higher risks of infectious morbidity and mortality [6, 7], diabetes [6] and stunting [1, 8, 10], leading to economic costs of approximately 302 billion US dollars per year globally [11]. These consequences of suboptimal breastfeeding may persist and play a role in perpetuating health and socioeconomic inequalities across generations. For example, stunting among women of reproductive age has been associated with increased risks of poor perinatal outcomes in their children [8], and lower intelligence is known to impact school and labor market performance [6, 8], which affects the individual’s socioeconomic status and that of his/her offspring.

Despite clear benefits, optimal breastfeeding practices have not been widely adopted. In 2016, UNICEF reported that, globally, only two-fifths of infants under 6 months of age received EBF and less than half (45%) were brought to their mothers for breastfeeding initiation within one hour after birth [1]. In LMICs, it has been reported that 37% of infants received EBF and less than 50% were breastfed within the first hour of their lives [6].

Suboptimal breastfeeding is a problem in Myanmar. The IYCF practices have been incorporated into the National Strategy since 2011 and the Baby Friendly Hospital Initiative (BFHI) has been implemented in Myanmar since 2010 [12]. Nevertheless, the country has not achieved its goals whereby 70% of infants are expected to receive EIBF [13] and 90% are expected to receive EBF [14]. The EBF prevalence seems to have increased over time yet it remains below than the target. In 2016, half (51%) of the infant population under the age of six months received EBF [15], compared to 23.6% in 2011 [16]. The average duration of EBF was 2.3 months in 2016 [15]. The EBF prevalence was relatively high in the first month and dropped dramatically as infants became older, falling to 38% when infants reached 4–5 months of age. EIBF, on the other hand, seems to have experienced a declining trend in Myanmar. The proportion of mothers who provided EIBF was 66.8% in 2015–2016 [15], compared to 75.8% in 2011 [17]. The low breastfeeding prevalence is reflected in poor child health, with neonatal, infant and under-five mortality being comparatively high at the rates of 23.1, 36.8 and 46.2 per 1000 live births respectively in 2018, according to the World Development Indicators database.

A deeper understanding of the mechanisms behind the decisions to provide EIBF and EBF is needed so that programs to encourage optimal breastfeeding practices can be more effectively designed. Existing studies from Myanmar have identified determinants of breastfeeding practices, which include the mother’s socioeconomic characteristics (e.g. area of residence, education, occupation, and economic status) as well as the extent to which the mother utilized maternal health services during the course of her pregnancy (e.g. number of antenatal care (ANC) visits, place of delivery and receipt of breastfeeding knowledge) [12, 18, 19]. The determinants that have been found to be statistically significant in Myanmar studies are consistent with those identified in studies from other LMICs [20–23]. Nevertheless, most Myanmar studies use region-specific (as opposed to nationally representative) data and focus on only EBF [12, 18, 19]. To fill the gap, this study aims to uncover determinants of both EBF and EBIF in Myanmar using the latest nationally representative survey collected in 2015–2016.

## Materials and methods

### Data source

This study used a cross-sectional study design. It used data from the first DHS survey in Myanmar: the 2015–2016 Myanmar Demographic and Health Survey (MDHS). A two-stage stratified cluster sampling method was used in the MDHS, using the 2014 Census as the sampling frame [24]. The first stage involved the identification of clusters, which in the MDHS referred to enumeration areas or ward/village tracts. The second stage involved the use of equal-probability systematic sampling to select households within each cluster. Given the response rate of 98%, the MDHS covered 12,500 households, whose information was collected during December 2015—July 2016 [15].

### Sample selection

The outcomes in the study, i.e. EIBF and EBF, were investigated using different samples. The exclusion criteria were (1) children outside the age cut-offs associated with EIBF and EBF in accordance with the WHO’s guidelines [2, 3], (2) children were deceased at the time of the interview and (3) children for whom information on the variables in the empirical model (explained below) was missing. The unit of analysis was the mother-child pair (from the same household).

The EIBF sample consisted of 1,506 observations, covering last-born children aged 0–23 months. The construction of the EIBF sample involved removing 48 and 115 observations of under-two children who had been deceased and who had missing information on variables in the model respectively.

**Fig 1:**
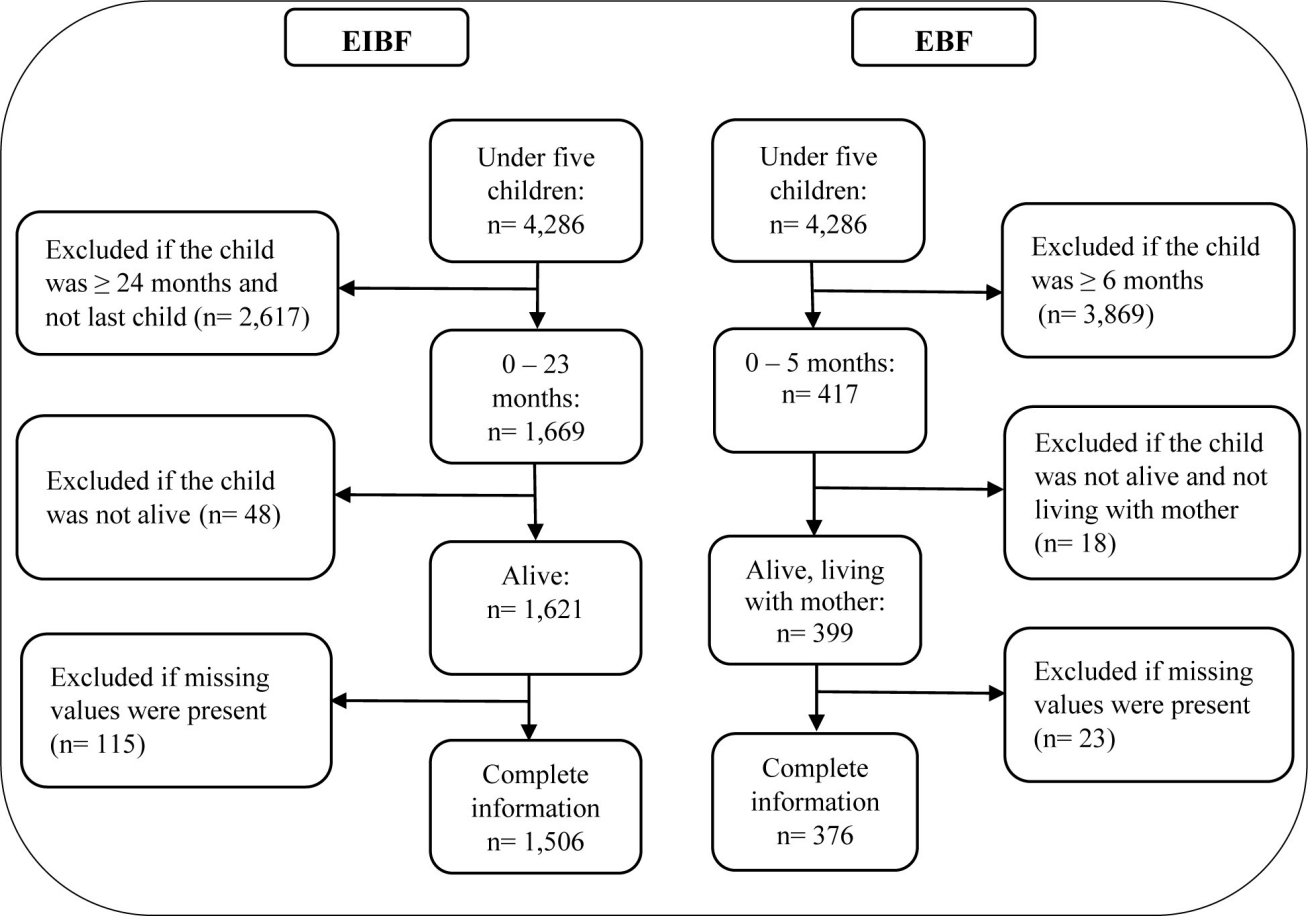
Inclusion and exclusion criteria of the early initiation of breastfeeding and the exclusive breastfeeding samples.

The EBF sample contained 376 observations, all of whom were children aged 0–5 months living with their mothers. Since the definition of EBF pertained to children younger than 5 months old, the construction of the EBF sample involved excluding 1,252 children who were 6–23 months old, 18 children who had been deceased or were not currently living with their mothers, and 23 children with missing information on variables included in the model. The step-by-step sample selection processes for both samples were illustrated in Fig 1.

### Outcome variables

Both EIBF and EBF were operationalized as binary variables. The EIBF variable was based on the question: *“How long after birth did you first put (name) to the breast*?”. It took the value of 1 if breastfeeding was initiated within the first hour, and 0 otherwise.

The EBF variable was based on the following questions: *“Are you still breastfeeding (name)*?*”*, *“Did (name) drink anything from a bottle with a nipple yesterday or last night (24 hours preceding the interview)*?*”*, and *“Any kind of milk (other than breast milk)*?*”*. It took the value of 1 if the infant received breast milk exclusively, and 0 otherwise.

### Explanatory variables

The explanatory variables were categorized into three groups, including bio-demographic, socio-economic and behavioral factors. The categorization was consistent with the framework used in existing studies [25, 26], and was adopted on the grounds that the referenced studies were based on Nigeria, which was an LMIC with socioeconomic settings that were comparable to Myanmar, and used data from a Demographic and Health Survey (DHS), similar to this study. The selection of the explanatory variables followed existing studies in Myanmar [12, 18, 19] and other LMICs, using DHS data [20–23]. It was also consistent with a broader conceptual framework developed by Rollins *et al*. [11]. This study included mother and infant attributes (individual factors), socioeconomic factors and behavioral factors (which addressed interactions between mothers and their health-related and work-related environments) as well as area-level (bio-demographic) variables, which partially captured differences in sociocultural attitudes and market factors faced by women in different geographical locations throughout the country.

All three groups of explanatory variables were hypothesized to be associated with EIBF and EBF. Bio-demographic factors referred to attributes of the mother, the child and the area of residence. Examples included age and gender of the child, the mother’s perception of the child’s birth size and the household’s region of residence. Socio-economic factors referred to household-level socioeconomic status indicators. They included the mother’s education and work status as well as the household’s economic status. Finally, behavioral factors referred to the pattern of maternal health services utilization by the mother. They encompassed ANC visits, mode and place of delivery, and postnatal checkup. The model for EBF included all of the above explanatory variables. In the EIBF model, certain variables—specifically, the child’s age and the receipt of postnatal checkup—were not accounted for because they were not related to the outcome, occurring *after* EIBF was determined.

### Statistical analysis

First, the data were explored. Descriptive statistics were presented as counts and percentages. Simple logistic regressions were performed, with each outcome being regressed on a single explanatory variable. Sampling weights were accounted for in order to ensure national representativeness. Results for the simple logistic regressions were expressed as crude odds ratios (COR), accompanied by 95% confidence intervals (CI). The bivariate analyses provided an implication as to whether a given explanatory variable was likely to be statistically significant in multiple logistic regressions and how alternative regression specifications could be formed.

Multiple logistic regressions were undertaken, taking into account sampling weights. The results were expressed as adjusted odds ratios (AOR) along with their 95% CIs. Three multiple logistic regression specifications, denoted as Model 1, Model 2 and Model 3, were explored. In Model 1, only bio-demographic factors were controlled for. In Model 2, socio-economic factors were added, and, in Model 3, all three groups of factors (including additionally behavioral factors) were included. The three models were compared, using likelihood ratio tests, and the model with the best fit was identified and discussed. Despite the fact that the simple logistic regressions (discussed below) suggested that some of the explanatory variables were not strong predictors of EBF and/or EIBF, this study included all explanatory variables anyway. The purpose was to ensure that the specifications were theoretically (as opposed to empirically) informed and that specification bias was reduced. It should also be noted that empirically-motivated specifications (whereby different combinations of variables that were statistically significant in the bivariate analyses were included in the multiple logistic model) produced results that were largely consistent with the preferred model below and therefore were not reported to conserve space.

Also, possible violations of the regression assumptions were considered. Multicollinearity was investigated using a simple correlation matrix. It was found that the variables had pairwise correlations that were low (below 0.6) and not statistically significant. Heteroskedasticity was tested using Breusch-Pagan tests, which provided evidence of heteroskedasticity at the 1% level for both EIBF and EBF. The problem was addressed through the use of Huber-White standard errors. All statistical analyses were carried out using Stata version 14.0.

### Ethical consideration

Ethics clearance was not sought since the study was based on secondary data that were publicly available on the DHS website.

## Results

### Descriptive statistics

Table 1 shows descriptive statistics of the outcome and the explanatory variables in the model. Among under-2 children in the EIBF sample (n = 1,506), 1,023 children or approximately 67.9% were breastfed within the first hour of birth. Children with the 2^nd^– 4^th^ birth rank had the highest proportion of EIBF, with 533 out of 774 of them (71.4%) having been breastfed within the first hour of birth. The EIBF prevalence varied across regions, with the highest rate in the hilly region and the lowest rate in the coastal region (72.6% versus 44.7%). Compared to the respective comparison groups, the EIBF prevalence was noticeably higher among children with the 2^nd^– 4^th^ birth rank (71.4%) and among mothers who had at least 4 antenatal care visits (70.7%) and those who had vaginal births (71.1%).

**Table 1:** Frequency and prevalence of EIBF and EBF by explanatory variables.

Variables	EIBF Sample (n = 1,506)		EBF sample (n = 376)	
	n (%), receiving EIBF	n, variable break-down	n (%) receiving EBF	n, variable break-down
**Outcome variables**				
**EIBF**				
Not within 1^st^ hour		483		
Within 1^st^ hour		1023		
**EBF**				
Non-EBF				180
EBF				196
**Bio-demographic factors**				
Child’s age (months)				
0–1			64 (72.0)	88
2–3			79 (53.4)	149
4–5			53 (38.2)	139
Sex of child				
Male	536 (66.3)	809	85 (41.9)	204
Female	487 (69.8)	697	111 (64.3)	172
Mother’s age (years)				
15–19	46 (74.2)	62	12 (54.8)	22
20–34	748 (67.5)	1108	148 (52.3)	283
35–49	229 (68.0)	336	36 (50.8)	71
Mother's perception of birth size				
Small	125 (61.3)	205	19 (30.2)	64
Average	633 (70.2)	901	120 (57.6)	209
Large	265 (66.1)	401	57 (54.7)	103
Birth order				
1^st^ rank	350 (63.3)	553	78 (51.8)	151
2^nd^– 4^th^ rank	553 (71.4)	774	98 (53.4)	184
> = 5^th^ rank	120 (66.9)	179	20 (47.8)	41
Place of residence				
Urban	274 (71.0)	385	52 (53.5)	97
Rural	749 (66.9)	1121	144 (51.7)	279
Region of residence				
Hilly	249 (72.6)	343	45 (54.2)	84
Coastal	69 (44.7)	153	17 (38.9)	43
Delta	375 (68.6)	548	68 (56.0)	121
Dry	330 (71.5)	462	66 (51.7)	128
**Socio-economic factors**				
Mother’s education				
No education	160 (70.3)	227	29 (44.8)	64
Primary	447 (67.8)	660	75 (49.9)	151
Secondary	332 (68.1)	487	64 (54.3)	18
> Secondary	84 (63.8)	132	28 (64.9)	43
Mother’s occupation				
Not working	456 (69.1)	660	96 (49.5)	193
Working	567 (67.1)	846	100 (54.9)	183
Economic status				
Poorest	266 (66.1)	402	51 (52.8)	96
Poorer	205 (64.3)	319	29 (40.9)	72
Middle	180 (70.2)	256	35 (54.6)	63
Richer	189 (69.1)	273	37 (50.8)	74
Richest	183 (71.6)	256	44 (61.9)	71
**Behavioral factors**				
ANC visits				
None	92 (57.97)	159	23 (44.1)	52
1–3	285 (65.8)	433	44 (45.6)	96
4 and above	646 (70.7)	914	129 (56.8)	228
Mode of delivery				
Caesarean section	188 (56.6)	332	53 (55.9)	96
Vaginal delivery	835 (71.1)	1174	143 (50.9)	280
Place of delivery				
Home	554 (69.3)	780	103 (51.1)	202
Health facility	469 (66.4)	706	93 (53.4)	174
Postnatal checkup				
Yes			71 (43.4)	163
No			125 (58.9)	213

(1) Sampling weights were accounted for in the estimation

(2) ANC = Antenatal care.

Among children aged 0–5 months in the EBF sample (n = 376), 196 children or approximately 52.2% were currently exclusively breastfed. Table 1 suggests that the highest EBF prevalence occurred among children aged 0–1 months and the lowest EBF prevalence was observed among children aged 4–5 months (72.0% versus 38.2%). The EBF prevalence was also highest among children who were perceived as having an average size at birth (57.6%), among female infants (64.3%), and among mothers who had not had a postnatal checkup (58.9%).

### Regression analyses

The results of simple logistic regressions in terms of COR are presented in S1 Table in the appendix. EIBF was found to be significantly and positively associated with the average perceived birth size, the 2^nd^– 4^th^ birth order, having had 4 or more ANC visits and having had a vaginal delivery. It was significantly and negatively associated with having residence in the coastal region. On the other hand, EBF was found to be significantly and positively associated with the fact that the infant was female and that he/she had average or large perceived birth sizes. It was also significantly and negatively associated with age of the child and the uptake of postnatal checkup. These results provided a benchmark against which robustness of the preferred multiple logistic regressions could be assessed. If the explanatory variables that were identified as statistically significant in the simple logistic regressions were indeed associated with the outcome variables, then they should remain statistically significant when more factors were controlled for in multiple logistic regressions.

As discussed in the Statistical Analysis sub-section, three different specifications, denoted as Models 1, 2 and 3, were explored. Model 1, containing only the bio-demographic factors, was nested completely within Model 2, which contained the bio-demographic and the socio-economic factors. Model 2 was nested completely within Model 3, where all three groups of factors were controlled for. Performed prior to the heteroskedasticity adjustment, likelihood ratio tests suggested that Model 3 provided a better fit than Model 2 (with the p-values of 0.000 for EIBF and 0.037 for EBF) as well as Model 1 (with the p-values of 0.000 for EIBF and 0.008 for EBF). Model 3 was therefore preferred. Results of Model 1 and 2 are shown in S2 Table in the Appendix. The results suggested that conclusions that may be drawn from this study were insensitive/ robust to specification changes.

Table 2 presents results of the preferred multiple logistic regression model (Model 3). The results were expressed in terms of AOR and 95% CIs based on heteroskedasticity-adjusted standard errors. The odds of EIBF were significantly and positively associated with the 2^nd^– 4^th^ birth order (AOR = 1.4), having had 4 or more ANC visits (AOR = 2.4), and having had a vaginal delivery (AOR = 2.5). The odds of EIBF were significantly and negatively associated with the coastal region (AOR = 0.3).

**Table 2:** Adjusted odds ratios from preferred multiple logistic regressions of EIBF and EBF.

Variables	EIBF [n = 1,506]		EBF [n = 376]	
	AOR [95%CI]	p-value	AOR [95%CI]	p-value
**Bio-demographic factors**				
Child’s age (months) (0–1 = reference category)				
2–3			0.3 [0.2–0.7]	0.002
4–5			0.2 [0.1–0.4]	0.000
Sex of child (Female)	1.2 [0.9–1.6]	0.120	3.0 [1.8–5.4]	0.000
Mother’s age (years) (15–19 = reference category)				
20–34	0.6 [0.3–1.3]	0.235	0.6 [0.2–2.0]	0.427
35–49	0.6 [0.3–1.4]	0.242	0.3 [0.1–1.3]	0.115
Perception of birth size (Small = reference category)				
Average	1.3 [0.9–2.0]	0.129	2.6 [1.2–5.4]	0.014
Large	1.1 [0.7–1.7]	0.683	2.5 [1.1–5.7]	0.034
Birth order (1^st^ rank = reference category)				
2^nd^– 4^th^ rank	1.4 [1.0–1.9]	0.035	1.5 [0.8–2.9]	0.205
> = 5^th^ rank	1.3[0.8–2.2]	0.334	1.7 [0.6–5.0]	0.336
Place of residence (Rural)	0.8 [0.6–1.2]	0.291	1.9 [0.8–4.4]	0.166
Region of residence (Hilly = reference category)				
Coastal	0.3 [0.2–0.5]	0.000	0.4 [0.2–1.1]	0.070
Delta	0.8 [0.5–1.1]	0.204	0.7 [0.3–1.4]	0.292
Dry	1.0 [0.7–1.5]	0.985	0.6 [0.3–1.2]	0.135
**Socio-economic factors**				
Mother’s education (No education = reference category)				
Primary	0.8 [0.5–1.2]	0.290	1.7[0.7–3.9]	0.230
Secondary	0.7 [0.4–1.2]	0.174	1.8 [0.7–4.7]	0.215
> Secondary	0.6 [0.3–1.2]	0.146	3.0[0.9–10.6]	0.081
Mother’s occupation (Working)	0.8 [0.6–1.1]	0.209	1.3 [0.7–2.2]	0.374
Economic status (Poorest = reference category)				
Poorer	0.8 [0.6–1.2]	0.335	0.4 [0.2–1.0]	0.041
Middle	1.1 [0.7–1.8]	0.557	0.9 [0.4–2.2]	0.844
Richer	1.1 [0.7–1.7]	0.813	0.9 [0.4–2.4]	0.876
Richest	1.3 [0.8–2.4]	0.309	1.4 [0.4–4.8]	0.545
**Behavioral factors**				
ANC visits (None = reference category)				
1–3	1.6 [1.0–2.5]	0.053	1.0 [0.3–2.5]	0.935
4 and above	2.4 [1.5–3.8]	0.000	1.9 [0.7–5.3]	0.187
Mode of delivery (Vaginal delivery)	2.5 [1.7–3.7]	0.000	0.8 [0.4–1.8]	0.603
Place of delivery (Health facility)	1.1 [0.7–1.5]	0.709	0.7 [0.3–1.5]	0.362
Postnatal checkup (Yes)			0.5 [0.3–0.9]	0.022

(1) AOR = adjusted odds ratios

(2) Robust standard errors were used in the calculation of the 95% CIs

(3) Sampling weights were accounted for in the estimation

(4) ANC = Antenatal care.

As for EBF, the preferred multiple logistic regression model showed that infants aged 2–3 months (AOR = 0.3) and 4–5 months (AOR = 0.2) were less likely to be breastfed exclusively compared with those aged 0–1 months. Being a female infant was associated with a 3 times higher likelihood of receiving EBF, compared to being male (AOR = 3.0). The odds of EBF were higher among mothers who perceived their babies to be of an average size (AOR = 2.6) or a large size (AOR = 2.5). Mothers that belonged to the poorer economic group had lower odds of EBF, compared to those belonged to the poorest group (AOR = 0.4). The receipt of a postnatal checkup was associated with lower EBF odds (AOR = 0.5). With the exception of economic status which was found to be statistically insignificant in the simple logistic regression for EBF, the results of simple and multiple logistic regression analyses for both EIBF and EBF were consistent.

## Discussion

Using the latest nationally representative survey and multiple logistic regression modeling, this study identified determinants of EIBF and EBF in Myanmar. The study was motivated partially by the high rates of infant mortality in the country as well as the rationale of the 1989 United Nations convention (whereby breastfeeding is considered a right, to which children are entitled, and the government is encouraged to provide access to optimal breastfeeding for children) [27].

Approximately 67.9% of the sample, consisting of under-2 children, received EIBF in 2015–2016. The EIBF prevalence in the sample differed from what was reported by the MDHS at 66.8% [15]. This was because, in the sample selection process, deceased children as well as those whose information on personal and household characteristics was incomplete were excluded. By international standards, the rate of almost 70% was considered satisfactory [28]. It was comparable with other countries with the same level of economic development, e.g. Namibia and Nepal [22, 29] and was higher than what was recently observed in Bangladesh (51%) [30]. The similarity in the practices of EIBF (and EBF) among LMICs was expected; determinants of breastfeeding practices, which included structural factors (i.e., sociocultural and market factors), the environment/settings in which one lived, and individual attributes [11], were likely to be quite similar among LMICs.

While EIBF was not significantly associated with the socio-economic factors, it was associated with some of the bio-demographic factors. Higher birth order was found to be positively associated with EIBF, consistent with an existing study conducted in Nigeria [25]. It is possible that multiparous mothers produce colostrum and breast milk earlier than nulliparous ones and are able to provide EIBF more easily [31]. Also, residing in the coastal region in Myanmar was found to be negatively associated with EIBF. Compared to other regions, the coastal region is more geographically dispersed, resulting in increased transportation difficulties, lack of obstetric care providers and lack of sustainability in the provision of neonatal care.

EIBF was also associated with some of the behavioral factors. First, caesarean section was found to be inversely associated with EIBF. A similar conclusion was reached in studies conducted in Ethiopia, Namibia and Turkey [21, 22, 32]. A possible explanation is that, as a procedure, caesarean section is more time-consuming and more complicated than vaginal delivery and mothers who undergo caesarean section may experience a higher degree of fatigue and are unable to breastfeed within the first hour [1].

Also, lack of or infrequent ANC (0–3) visits was inversely associated with EIBF. The importance of ANC visits for EIBF is mixed in the literature, with positive effects found in studies based in South Asia, Nigeria and Nepal [23, 25, 29], and negative effects documented in a study based in Namibia [22]. The positive impact of ANC visits on EIBF observed here may be explained by the fact that, through ANC visits and interactions with health care professionals, mothers may learn to better appreciate the benefits of optimal breastfeeding practices and therefore adopt EIBF.

In order to promote EIBF in Myanmar, maternal health services need to be strengthened. Given that caesarean section was found to be inversely associated with EIBF, it is suggested that causes of redundant caesarean delivery be identified, specific guidelines and indications for caesarean section be strengthened, and women be encouraged to take the vaginal delivery option whenever possible. Also, it is suggested that the Baby Friendly Hospital Initiative (BFHI) program, which is already implemented in Myanmar, be scaled up. In particular, as existing studies have found a positive dose-response relationship between BFHI and breastfeeding practices [33], the number of baby-friendly hospitals should be expanded and health professionals should be provided with on-the-job training as to how to inform mothers about the benefits of EIBF and how to effectively provide breastfeeding support [27, 28].

With regard to EBF, this study found that 52.2% of infants aged 0–5 months in the sample received exclusive breastfeeding in 2015–2016. The figure was consistent with the MDHS report [15] if/when the same age group was considered. It was also in line with the minimum recommended WHO’s target of 50%, but was lower than the target of 90% set by the country itself [14, 34]. Also, the rate was found to be higher than what was discovered in a 2015 study based in one region in Myanmar (15%) [18], higher than what was found in a Nigerian study (16.4%), and lower than a Nepalese study conducted in 2011 (66.3%) [20, 35]. The comparison suggested that there was a wide variation of EBF rates even among LMICs.

EBF was found to be associated with some of the bio-demographic factors. First, age of the child was negatively associated with EBF, consistent with existing studies from other countries [35–37]. This likely reflects the general misbelief that breast milk alone cannot provide sufficient nutrients for infant growth and development [12]. It suggests that there is a need to inform mothers and caregivers, through a public platform (including, for example, ANC visits), about advantages of EBF and to assure them that breast milk is sufficient for babies up to six months of age.

EBF was found to be significantly related with sex and the perceived birth size of the child. In this study, female infants were found to have a higher chance of receiving EBF compared with male infants. A similar conclusion was reached in studies conducted in Nigeria and Vietnam [20, 36], yet a study in India suggested that male infants were more likely to receive EBF [23]. The difference may stem at least partially from cultural norms, which dictate certain gender preference, that vary across countries. Also, this study showed that infants perceived as having a large size at birth (and correspondingly a higher perceived likelihood of infant survival) were more likely to receive EBF, consistent with studies conducted in South Asia and India [23, 37].

Unlike EIBF, EBF was found to be significantly associated with one socioeconomic factor, i.e., economic status of the household. It was found that mothers that belonged to poorer households had a lower probability of providing EBF, compared to those in the poorest category as well as richer households. Evidence for the association between economic status and EBF was nevertheless mixed in the literature [12, 18, 19, 38].

Finally, it was found that the receipt of postnatal checkup, as one of the behavioral factors, reduced the probability of EBF. This finding was consistent with a study conducted in Indonesia [38]. The negative association may be explained by the fact that children who do not receive EBF are more likely to have health problems (e.g. diarrhea) and may be more likely to seek postnatal care.

In order to promote EBF in Myanmar, campaigns to encourage mothers to provide EBF, regardless of socioeconomic status or gender and size of the child, should be considered. Moreover, given that EBF was positively associated with larger perceived size of infants, programs for pregnancy diet and nutrition, e.g. nutrition counseling and prenatal meal planners, should be developed. Impacting health and growth of infants and consequently their perceived sizes, pregnancy diet and nutrition programs should be implemented at the community level so that differences in community-specific food cultures can be accounted for [8].

This study made at least two contributions. First, based on the latest MDHS and accounting for survey weights, the results were nationally representative and the conclusions should be generalizable in the context of Myanmar. Second, this study represented the first nationwide study to investigate both EIBF and EBF practices.

Limitations existed in the study. First, since the study design was cross-sectional, it was not possible to address causality between the outcome variables and the explanatory variables. Also, since the MDHS data in this study were collected retrospectively, potential recall bias might have been present due to memory decay of the respondents. There was also a possibility of social desirability bias because all answers concerning breastfeeding practices by the mothers were self-reported and some of the questions may be deemed culturally sensitive, impacting the accuracy of the responses.

## Conclusions

Myanmar needs to improve the nutritional status of children and reduce infant and under-five mortality rates. The prevalence of optimal breastfeeding practices is currently below the national targets. This study identified a number of important determinants of both EIBF and EBF. As for EIBF, it was found that prior utilization of maternal and child health services was associated with an increased likelihood of EIBF. The implication is that maternal and child health services should be scaled up [28], as they play a crucial role in improving the nutrition level and wellbeing of children. In particular, the number of baby-friendly hospitals should be expanded and health professionals should be provided with on-the-job training and evaluated to ensure the quality of services regarding breastfeeding support [28]. Specific guidelines and indications for caesarean section should also be strengthened since unnecessary caesarean sections are inversely associated with EIBF. As for EBF, this study found that EBF varied with perception of mothers regarding the size of their infants. Community health education programs on pregnancy diet and nutrition, which impact health and growth of the infant [8], are likely to be crucial for the promotion of EBF.

## Supporting information

S1 Table Crude/unadjusted odds ratios from simple logistic regressions of EIBF and EBF. (PDF)

S2 Table Adjusted odds ratios from Model 1 and Model 2 of EIBF and EBF. (PDF)
