# Supplementary material for: Determinants of breastfeeding practices in Myanmar: Results from the latest nationally representative survey
Source: PLoS One. 2020 Sep 24;15(9):e0239515. doi: 10.1371/journal.pone.0239515 (PMC7514058; doi:10.1371/journal.pone.0239515)
Supplement: S1 Table — (PDF) [file pone.0239515.s001.pdf]

S1 Table. Crude/unadjusted odds ratios from simple logistic regressions of EIBF and EBF

Variables	EIBF [n=1,506]	EBF [n=376]
	COR [95%CI]	COR [95%CI]
**Bio-demographic factors**		
Child's age (months) (0-1 = base category)		
2-3		0.4**
		[0.2 - 0.8]
4-5		0.2***
		[0.1 - 0.5]
Sex of child (Female)	1.2	2.5***
	[0.9 - 1.5]	[1.6 – 3.9]
Mother's age (years) (15-19 = base category)		
20-34	0.7	0.9
	[0.4 - 1.4]	[0.3 - 2.5]
35-49	0.7	0.8
	[0.4 - 1.5]	[0.3 – 2.6]
Perception of birth size (Small = base category)		
Average	1.5*	2.9**
	[1.0 - 2.1]	[1.6 – 5.7]
Large	1.2	2.7**
	[0.8 - 1.8]	[1.3 - 5.5]
Birth order (1st rank = base category)		
2nd – 4th rank	1.5**	1.1
	[1.1 - 1.9]	[0.7 - 1.8]
>=5th rank	1.2	0.9
	[0.8 - 1.8]	[0.4 – 1.9]
Place of residence (Rural)	0.8	0.9
	[0.6 - 1.1]	[0.5 – 1.6]
Region of residence (Hilly = base category)		
Coastal	0.3***	0.5
	[0.2 - 0.5]	[0.3 - 1.1]
Delta	0.8	1.1
	[0.6 - 1.2]	[0.6 – 2.0]
Dry	0.9	0.9
	[0.7 - 1.4]	[0.5 - 1.7]
**Socio-economic factors**		
Mother's education (No education = base category)		
Primary	0.9	1.2
	[0.6 - 1.3]	[0.6 – 2.4]
Secondary	0.9	1.4
	[0.6 - 1.3]	[0.7 – 2.9]
> Secondary	0.7	2.1

	[0.4 - 1.2]	[0.8 – 5.6]
Mother's occupation (Working)	0.9<br>[0.7 - 1.2]	1.2<br>[0.7 – 1.9]
Economic status (Poorest = base category)		
Poorer	0.9<br>[0.6 - 1.2]	0.6<br>[0.3 - 1.2]
Middle	1.2<br>[0.8 - 1.8]	1.1<br>[0.5 - 2.2]
Richer	1.4<br>[0.9 - 1.7]	0.9<br>[0.4 – 1.8]
Richest	1.3<br>[0.9 - 1.9]	1.4<br>[0.7 – 2.9]
**Behavioral factors**		
ANC visits (None = base category)		
1 – 3	1.4<br>[0.9 - 2.2]	1.1<br>[0.4 - 2.2]
4 and above	1.8**<br>[1.2 - 2.6]	1.6<br>[0.8 – 3.3]
Mode of delivery (Vaginal delivery)	1.9***<br>[1.4 - 2.6]	0.8<br>[0.5 - 1.4]
Place of delivery (Health facility)	0.9<br>[0.7 - 1.1]	1.1<br>[0.7 - 1.7]
Postnatal checkup (Yes)		0.5**<br>[0.3 - 0.8]

Notes: (1) COR = crude odds ratios;
(2) *p < 0.05, **p < 0.01, ***p< 0.001;
(3) Robust standard errors were used in the calculation of the 95% CIs;
(4) Sampling weights were accounted for in the estimation.
