# Supplementary material for: Determinants of breastfeeding practices in Myanmar: Results from the latest nationally representative survey
Source: PLoS One. 2020 Sep 24;15(9):e0239515. doi: 10.1371/journal.pone.0239515 (PMC7514058; doi:10.1371/journal.pone.0239515)
Supplement: S2 Table — (PDF) [file pone.0239515.s002.pdf]

S2 Table. Adjusted odds ratios from Model 1 and Model 2 of EIBF and EBF

Variables	EIBF [n=1,506]		EBF [n=376]	
	Model 1	Model 2	Model 1	Model 2
	AOR [95%CI]	AOR [95%CI]	AOR [95%CI]	AOR [95%CI]
**Bio-demographic factors**				
Child's age (months) (0-1 = base category)				
2-3			0.4**	0.3**
			[0.2 - 0.8]	[0.2 - 0.6]
4-5			0.2***	0.2***
			[0.1 - 0.4]	[0.1 - 0.4]
Sex of child (Female)	1.2	1.2	2.7***	3.0***
	[0.9 - 1.6]	[0.9 - 1.6]	[1.6 – 4.6]	[1.8 - 5.2]
Mother's age (years) (15-19 = base category)				
20-34	0.6	0.6	1.0	0.7
	[0.3 - 1.2]	[0.3 - 1.2]	[0.3 – 3.1]	[0.2 – 2.3]
35-49	0.5	0.6	0.6	0.4
	[0.3 - 1.1]	[0.3 - 1.2]	[0.2 – 2.3]	[0.1 - 1.6]
Perception of birth size (Small = base category)				
Average	1.3	1.4	2.7**	2.7*
	[0.9 – 1.9]	[1.0 – 2.0]	[1.3 – 5.5]	[1.3 – 5.9]
Large	1.1	1.2	2.7*	2.7*
	[0.8 - 1.7]	[0.8 - 1.8]	[1.2 - 5.8]	[1.2 – 6.0]
Birth order (1st rank = base category)				
2nd – 4th rank	1.6**	1.5**	1.2	1.5
	[1.2 – 2.1]	[1.1 – 2.0]	[0.7 – 2.2]	[0.8 – 2.8]
>=5th rank	1.4	1.3	1.1	1.5
	[0.9 – 2.3]	[0.8 - 2.2]	[0.4 – 3.1]	[0.5 – 4.7]
Place of residence (Rural)	0.8	0.9	0.9	1.7
	[0.6 - 1.2]	[0.6 - 1.3]	[0.5 – 1.7]	[0.8 – 3.8]
Region of residence (Hilly = base category)				
Coastal	0.3***	0.3***	0.4	0.4*
	[0.2 - 0.5]	[0.2 - 0.5]	[0.2 - 1.0]	[0.2 - 1.0]
Delta	0.8	0.8	0.9	0.7
	[0.6 - 1.2]	[0.6 - 1.2]	[0.6 – 1.8]	[0.3 - 1.4]
Dry	1.0	1.0	0.7	0.6
	[0.7 - 1.4]	[0.7 - 1.4]	[0.4 - 1.4]	[0.3 - 1.1]
**Socio-economic factors**				
Mother's education (No education = base category)				
Primary		0.9		1.5
		[0.6 - 1.3]		[0.6 – 3.4]
Secondary		0.8		1.9
		[0.5 - 1.3]		[0.8 - 4.8]
> Secondary		0.6		3.2

	[0.3 - 1.1]	[0.9 – 11.1]
Mother's occupation	0.8	1.3
(Working)	[0.7 - 1.1]	[0.8 - 2.2]
Economic status (Poorest = base category)		
Poorer	0.9	0.5
	[0.6 - 1.2]	[0.2 - 1.0]
Middle	1.1	1.0
	[0.7 - 1.8]	[0.4 - 2.3]
Richer	1.1	0.9
	[0.7 - 1.7]	[0.4 - 2.3]
Richest	1.3	1.5
	[0.8 - 2.3]	[0.5 - 4.8]

Notes: (1) AOR = adjusted odds ratios;

(2) *p < 0.05, **p < 0.01, ***p< 0.001;

(3) Robust standard errors were used in the calculation of the 95% CIs;

(4) Sampling weights were accounted for in the estimation.
